# Experimental and Numerical Study on the Impact Response of Composite Sandwich Structures with Different Cores

**DOI:** 10.3390/polym16233436

**Published:** 2024-12-07

**Authors:** Guangshuo Feng, Chunlu Xiao, Bo Liu, Haitao Zhang, Peipei Jia, Caizheng Wang

**Affiliations:** 1 School of Mechanical Engineering, University of Science and Technology Beijing, Beijing 100083, China; fengguangshuo@ustb.edu.cn (G.F.); liubo1@ustb.edu.cn (B.L.); 2 ShenSi Lab, Shenzhen Institute for Advanced Study, University of Electronic Science and Technology of China, Shenzhen 518110, China; xiaochunlu@std.uestc.edu.cn (C.X.); zhanght23@uestc.edu.cn (H.Z.)

**Keywords:** sandwich structure, impact experiment, finite element simulation, failure process

## Abstract

This study analyzes the impact mechanical response of sandwich structures with foam and wood cores through experimental and numerical methods. The aim is to determine whether a sustainable core material, such as cork wood, can serve as a reliable alternative to the commonly used Polystyrene (PS) foam core in sandwich structures. Impact experiments were conducted at varying energy levels using an INSTRON CEAST 9350 drop tower, demonstrating the superiority of sandwich structures compared to single-material alternatives. Numerical models were developed in ABAQUS, where glass fiber reinforced polymer (GFRP) composite panels were represented using solid element C3D8R and the 3D Hashin failure criteria, which were incorporated via the user subroutine VUMAT. The results indicate that the contact force of the sandwich structure with a wood core surpassed that of the foam core counterpart. In both sandwich structures, damage initially occurred at the impact point on the surface, leading to plastic deformation and damage within the core, while the composite panel on the rear surface ultimately failed. These findings provide valuable insights for designers, enabling parametric studies to select appropriate core materials that enhance the impact resistance of sandwich structures.

## 1. Introduction

Composite materials with high specific stiffness and strength have been commonly used in lightweight structures. The sandwich structure consists of two composite panels and a core [1], which is one of the most promising composite structures in the aerospace and transportation industries [2,3,4]. Although these composite structures exhibit good in-plane properties, they are vulnerable to accidents and impact events during assembly, maintenance, and service [5,6,7]. Barely visible damage can significantly reduce the bearing capacity of composite structures and may lead to catastrophic failure in the service cycle. Therefore, it is of great significance to study the dynamic response and failure mechanisms of composite sandwich structures under impact loads.

The impact behaviors of sandwich structures with different cores [8,9] have been widely reported in the literature, such as corrugated cores [10], honeycomb cores [11,12], and lattice cores [13,14]. Due to the existence of the core, the impact response of composite sandwich structures is different from that of composite laminates. Moreover, the mechanical behavior of sandwich structures is also strongly dependent on the geometry used for the face-sheet and cores [15,16]. Sun et al. [17] investigated the dynamic response of multilayer sandwich beams with foam-filled trapezoidal corrugated and foam cores subjected to impact from heavy masses, employing both analytical and numerical methods. Their theoretical model provided a detailed discussion on the effects of impact position, foam strength, and face-sheet thickness on the dynamic response of these multilayer sandwich beams. Mocianet al. [18] studied the impact behavior of foam-cored sandwich structures with aluminum and GFRP panels, and conducted a detailed analysis of the energy absorption and damage characteristics of the sandwich structure. The results showed that the energy absorption performance of the sandwich panel was affected by the initial impact velocity on the one hand and the type of panel and core material on the other hand. Zhu et al. [19] studied the failure mode of sandwich structures under static indentation and impact by theoretical, experimental, and numerical methods. The results showed that panel thickness had a great influence on both failure mode and ultimate load, while the density of the sandwich structure only had an influence on the final failure mode. Park et al. [20] analyzed the impact history of sandwich structures and concluded that the impact response was significantly influenced by core thickness and varied depending on the panel materials used. In addition, it is necessary to acknowledge the importance of using sustainable core and face-sheet materials [21]. Wang et al. [22] experimentally compared the mechanical response of several sandwich panels with different core materials. The results showed that sandwich plates with cork cores absorbed the least amount of energy and also exhibited less damage than the other sandwich samples for higher load conditions.

The damage patterns, failure characteristics, and impact response curves of sandwich structures under impact can be obtained from experimental methods. However, both the panels and core materials of sandwich structures are made of different materials due to practical use. The combinations of panels and cores are also complex and diverse, which increases the difficulty of experiments. Numerical analysis can analyze the damage process of the sandwich structure and showed a very positive effect on the application of the structure. Huo et al. [23] simulated the impact response of the foam core sandwich plate by a full-size finite element model and established an initial peak impact load analysis model based on the energy theory. The analysis results were in good agreement with the experimental and numerical results. Mahdian et al. [24] conducted a numerical investigation on the impact of steel wire-reinforced foam core/composite skin sandwich panels, achieving good agreement between numerical and experimental results. Zhang et al. [25] studied the impact resistance of a honeycomb sandwich structure subjected to impact by numerical and experimental methods. Physically based Puck’s composite failure criteria were performed to obtain interlaminar damage initiation. The results showed that various damage modes occurred, and, also, a delamination area in the face-sheet arose from increasing the impact energy. Chen et al. [26] established a numerical model addressing the intralaminar damage, interlaminar and adhesive delamination, and strain rate effect of the materials to predict the damage behaviors of composite sandwich structures with a honeycomb core subjected to perforation impact. The numerical results showed good agreement with experimental results in key perforation mechanisms and associated damage patterns. Pan et al. [27] established the finite element (FE) model to analyze the response process of the structure and to explore the effect of staggered angles on the deformation and load-carrying capacity. The results showed that, the smaller the staggered angle of two corrugated cores, the higher the peak impact force of the structure. The core damage mode of sandwich structures with small, staggered angles changed from local buckling to global bearing with the increase in impact energy. The impact damage of composite sandwich structures may occur on the panel, core material, and panel/core interface. Typical failure modes of the panel/core mainly contain intralaminar (fiber breakage, matrix cracking) and interlaminar (delamination) damage [28,29]. These damage behaviors depend on various factors including impact energy, material properties, geometric parameters, and boundary conditions.

In this study, the mechanical responses of composite sandwich structures with cork wood and Polystyrene (PS) foam cores were analyzed using experiments and numerical simulations to assess whether sustainable core materials like cork wood can reliably replace the commonly used Polystyrene (PS) foam core [30]. A 3D Hashin failure model of the composite material was embedded into ABAQUS by user subroutine VUMAT. The effects of the core material on the mechanical response and damage process of different sandwich structures were analyzed by numerical methods. Comparing the results with those of single-layer panels can help reveal the extent to which sandwich structures can enhance impact resistance and energy absorption, providing designers with valuable design references and insights for selecting core materials in sandwich structures, particularly sustainable core materials.

## 2. Experimental Method

### 2.1. Material and Fabrication

**Figure 1:**
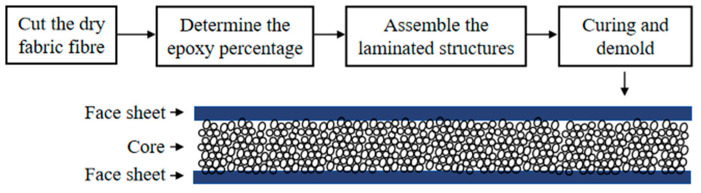
The process of preparing sandwich structures.

The glass fiber reinforced epoxy matrix laminates were manufactured by a wet layup process for the sandwich panels. The fiber/resin weight ratio deduced from the dry weight of the fabric and the final weight of the cured samples was approximately 60:40. The dimensions of the square plates were fixed as 150 mm × 150 mm. Six layers of plain woven glass fabric with the ply orientation [0/45/0]_s_ were used. The two face-sheet laminates of glass fiber were bonded on each side of the core material, and the sandwich panel was co-cured at room temperature under a vacuum bag for 24 h to ensure good adhesion. The nominal thickness of the cured sandwich panels was 14 mm, with a 10 mm thick core and 2 mm thick face-sheets. This procedure is as follows: ① Cut the dry glass fiber fabric larger than the core material, in the orientation as mentioned above; ② Weigh the fiber to determine the amount of epoxy resin needed; ③ One face-sheet is laid on a bench, and the core material is placed on top of the sheet. The process is repeated for the other face-sheet; ④ The panel is then placed in the vacuum machine with a weight added on top to cure for 24 h (Figure 1).

### 2.2. Low-Velocity Impact Test

**Figure 2:**
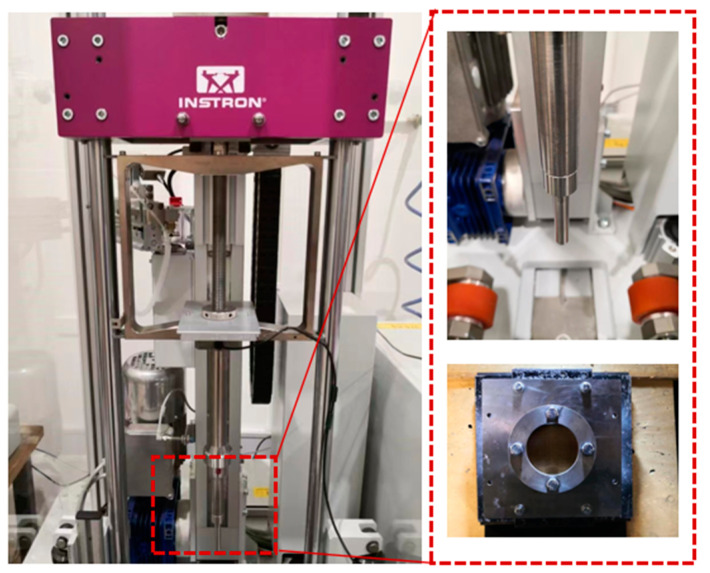
The drop tower equipment (**left**: falling carriage system; **right**: impactor and support fixture).

An INSTRON CEAST 9350 drop tower was used to conduct the impact tests presented in accordance with the requirements of ASTM D7136 [31]. The general features of the machine and data acquisition system are shown in Figure 2. The drop tower is equipped with a free-falling carriage system that includes an impactor and a load cell. The system operates on the principle of energy conservation, balancing potential and kinetic energy by varying the drop height of the carriage, and has the option of adding additional mass to the system. The system can simulate drop heights of upwards of 30 m by accelerating the carriage via springs at the top of the system. The square tests specimens were subjected to a concentrated impact by using a 16 mm diameter hemispherical striker with 5.5 kg of total weight from various heights. During the test, the impactor strikes the center of the square target plate, which is clamped with a solid steel impact support fixture with an internal diameter of 135 mm. To prevent the rebounding of the specimen during the impact event, an anti-rebound system that catches the impactor was also implemented to prevent the striker from reloading the specimen.

All the experiments included the single GFRP plate with 20 J, 40 J, 60 J, 80 J, and 100 J energy impact tests and sandwich structures (with PS foam and cork wood cores) with 100 J impact tests.

## 3. Finite Element Modelling

### 3.1. Damage Model of Composite Panel

#### 3.1.1. Damage Initiation Criteria

Low-velocity impacts are out-of-plane loadings that involve the interaction between shear stress components and principal stress components. To predict the in-plane and out-of-plane damage of composite panels in sandwich structures, the 3D Hashin criteria, considering fiber tension damage, fiber compression damage, matrix tension damage, and matrix compression damage, are used. The detailed expression is as follows:

Fiber tension damage ($\sigma_{11}\geq 0$)

(1) $$F_{ft}={\left(\frac{\sigma_{11}}{S_{ft}}\right)}^{2}+{\left(\frac{\sigma_{12}}{S_{12}}\right)}^{2}{+\left(\frac{\sigma_{13}}{S_{13}}\right)}^{2}$$

Fiber compression damage ($\sigma_{11}<0$)

(2) $$F_{fc}={\left(\frac{\sigma_{11}}{S_{fc}}\right)}^{2}$$

Matrix tension damage ($\sigma_{22}+\sigma_{33}\geq 0$)

(3) $$F_{mt}={\left(\frac{\sigma_{22}}{S_{mt}}\right)}^{2}$$

Matrix compression damage ($\sigma_{22}+\sigma_{33}<0$)

(4) $$F_{mc}=\left[{\left(\frac{\sigma_{22}}{{2S}_{23}}\right)}^{2}-1\right]\frac{\sigma_{22}+\sigma_{33}}{S_{mt}}+{\left(\frac{\sigma_{22}+\sigma_{33}}{2S_{23}}\right)}^{2}+\frac{\sigma_{12}^{2}+\sigma_{13}^{2}}{S_{12}^{2}}+\frac{\sigma_{23}^{2}-\sigma_{22}\sigma_{33}}{S_{23}^{2}}$$

**Figure 3:**
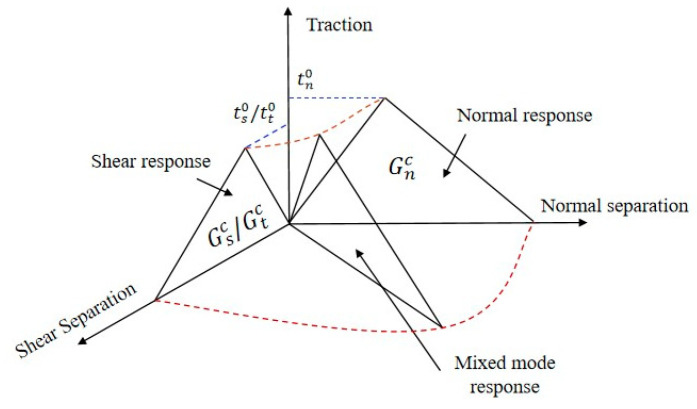
Diagram of cohesive zone model.

To investigate the interface damage of a composite panel, the cohesive zone model is used (Figure 3). The model provides a relationship between cracks length and fracture toughness in the normal direction and shear direction as follows:

Interface damage in the normal direction ($\sigma_{33}\geq 0$)

(5) $$F_{it}={\left(\frac{\sigma_{33}}{S_{it}}\right)}^{2}+{\left(\frac{\tau_{13}}{S_{13}}\right)}^{2}+{\left(\frac{\tau_{23}}{S_{23}}\right)}^{2}$$

Interface damage in the shear direction ($\sigma_{33}<0$)

(6) $$F_{ic}={\left(\frac{\tau_{13}}{S_{13}}\right)}^{2}+{\left(\frac{\tau_{23}}{S_{23}}\right)}^{2}$$

in which $\sigma_{11}$, $\sigma_{22}$, and $\sigma_{33}$ were stress components corresponding to the fiber axial direction, in-plane fiber transverse direction, and out-of-plane direction. $\tau_{12}$, $\tau_{13}$, and $\tau_{23}$ were shear stress components. $S_{ft}$ and $S_{fc}$ were the tension and compression strength of the fiber; $S_{mt}$ and $S_{mc}$ were the tension and compression strength of the matrix; $S_{it}$ was the interface delamination strength in the normal direction; $S_{12}$,${\;S}_{13},$ and $S_{13}$ were the shear strength.

#### 3.1.2. Damage Evolution

Equivalent strain damage evolution methods based on fracture energy are used to describe the damage evolution as:

(7) $$d_{i}=\frac{\varepsilon_{eq,i}^{f}\left(\varepsilon_{eq,i}-\varepsilon_{eq,i}^{0}\right)}{\varepsilon_{eq,i}\left(\varepsilon_{eq,i}^{f}-\varepsilon_{eq,i}^{0}\right)}$$

with $\varepsilon_{eq,i}^{0}=S_{i}/E_{0,j}$, $\varepsilon_{eq,i}^{f}=2G_{i}/\left(S_{i}l_{c}\right)$

in which $\varepsilon_{eq,i}^{0}$ and $\varepsilon_{eq,i}^{f}$ represented the initial and final failure strain, respectively; $S_{i}$ was the specific strength; $G_{i}$ was the fracture energy; $E_{0,j}$ was the modulus; $l_{c}$ was the characteristic length of the element, which was calculated as the cube root of the element volume.

Each composite lamina was considered as a transversely isotropic material, and its damaged stress–strain relationship can be expressed as $\sigma_{d}=C_{d}\varepsilon$. The degraded stiffness matrix $C_{d}$ was expressed as follows [19]:

$$C_{d}=\frac{1}{∆}\left[\begin{array}{cc} \begin{array}{ccc} d_{f}E_{11}\left(1-d_{m}\nu_{23}\nu_{32}\right) & d_{f}d_{m}E_{11}\left(\nu_{21}+\nu_{23}\nu_{31}\right) & d_{f}E_{11}\left(\nu_{31}+d_{m}\nu_{21}\nu_{32}\right)\\ & d_{m}E_{22}\left(1-d_{f}\nu_{13}\nu_{31}\right) & d_{m}E_{22}\left(\nu_{32}+d_{f}\nu_{12}\nu_{31}\right)\\ & & {d_{i}E}_{33}\left(1-d_{m}\nu_{12}\nu_{21}\right) \end{array} & \\ & \begin{array}{ccc} \Delta d_{f}d_{m}G_{12} & & \\ & \Delta d_{i}d_{m}G_{23} & \\ & & \Delta d_{f}d_{i}G_{13} \end{array} \end{array}\right]$$

with

(8) $$\left\{\begin{array}{c} \begin{array}{c} d_{f}=\left(1-d_{ft}\right)\left(1-d_{fc}\right)\\ d_{m}=\left(1-d_{mt}\right)\left(1-d_{mc}\right) \end{array}\\ \begin{array}{c} d_{i}=\left(1-d_{it}\right)\left(1-d_{ic}\right)\\ ∆=1-d_{f}d_{m}\nu_{12}\nu_{21}-d_{m}\nu_{23}\nu_{32}-d_{f}\nu_{13}\nu_{31}-2d_{f}d_{m}\nu_{12}\nu_{32}\nu_{13} \end{array} \end{array}\right.$$

in which $d_{f}$, $d_{m}$, and $d_{i}$ were the damage variables for fiber, matrix, and interface, respectively. The subscripts t and c represented the tension and compression loading condition.

### 3.2. Finite Element Implementation

**Figure 4:**
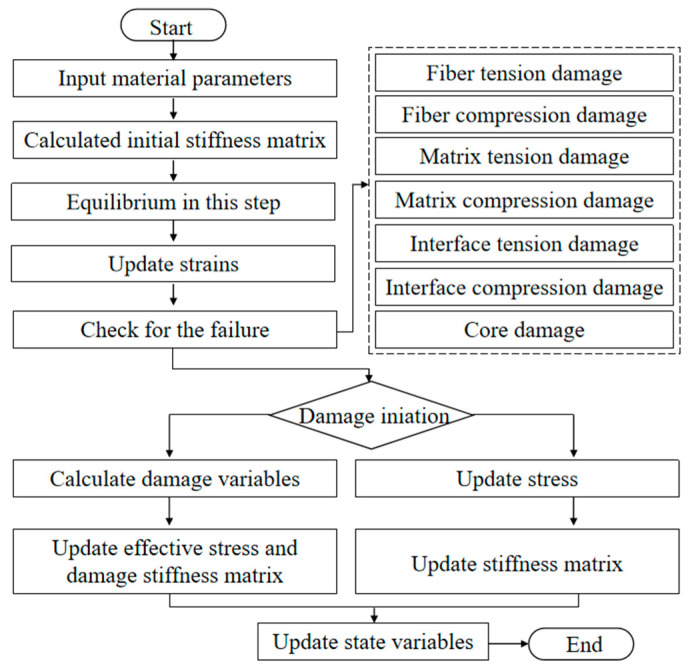
Flowchart of finite element simulation with user subroutine.

The established damage initiation and evolution model were implemented in the finite element software ABAQUS SIMULIA6.12 by user subroutine VUMAT. The process is described as follows (Figure 4). The mechanical parameters of the composite material are input to capture the constitutive relationships of the composite materials. The established model is then translated into a user-defined Fortran subroutine, and the strain and stress components are calculated. The VUMAT subroutine requires element deletion if one of the failure criteria is satisfied. Once all element stresses and strains are set to zero, the deletion flag is activated.

### 3.3. Finite Element Model

**Figure 5:**
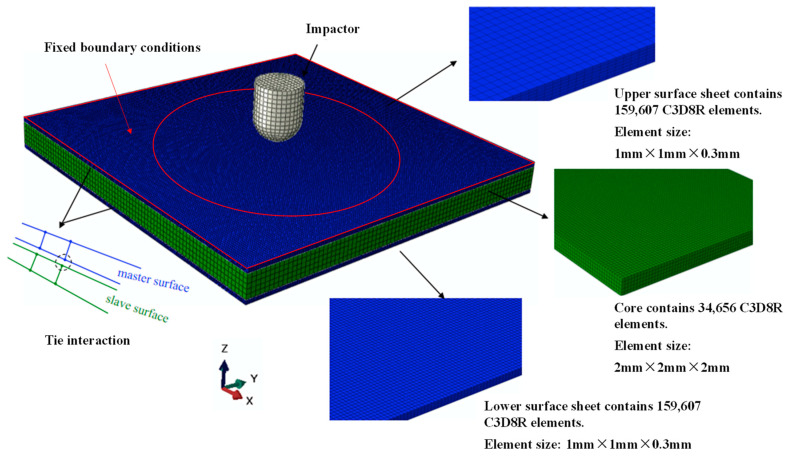
Finite element model of sandwich structure under impact loading.

The low-velocity impact simulation is conducted by the finite element software ABAQUS. Although the experimental process is only a few milliseconds, the finite element simulation may take dozens of hours. Considering the efficiency and accuracy of numerical simulation, this paper adopts the finite element mesh model shown in Figure 5. The geometry dimension is modeled according to the specimen design in Section 2. The number and quality of meshes affect the simulation time and large cell distortion deformations. The meshes are divided using intermediate local encryption. The composite panel and cores are separately modeled with 159,607 and 34,656 solid elements C3D8R. Tie interaction is applied to model the connection between panels and core. The boundary conditions were set with constraints by prescribing a fixed displacement for nodes outside the circular area diameter, i.e., all nodes outside 135 mm in the sandwich plate to be fixed. The impactor is modeled as a rigid body with a diameter of 16 mm.

It must be noted that, since the focus of this paper is on dynamic performance, no separate quasi-static tests have been conducted. The mechanical properties of the materials discussed in this paper are sourced from the references [22,32,33,34,35,36], as shown in the Table 1 below.

## 4. Results and Discussion

### 4.1. Impact Experimental Results of Simple GFRP

#### 4.1.1. Contact Force and Tip Displacement Histories

**Figure 6:**
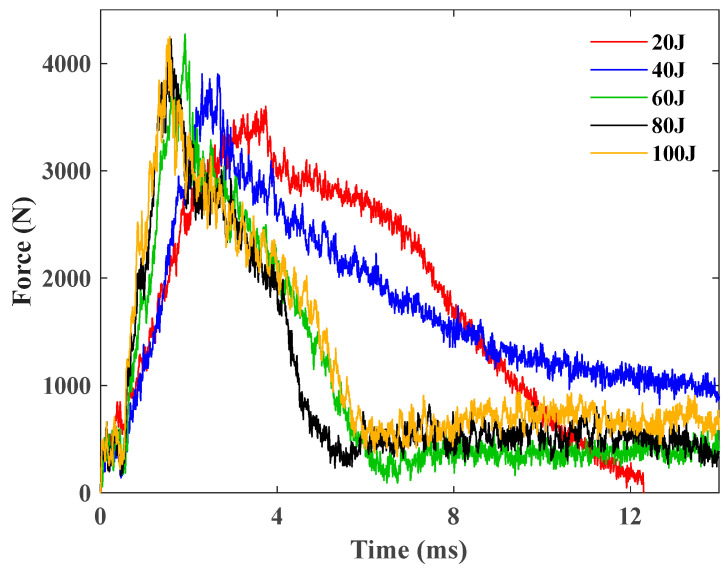
Contact force histories of the GFRP sample under 20 J, 40 J, 60 J, 80 J, and 100 J.

The raw data obtained from the drop hammer impact device are expressed as a time axis of the contact force, i.e., the correlation of the contact force between the impactor and the samples over time. Under the impact of a 5.5 kg impactor, the contact force histories of the GFRP sample (face-sheets) under different energies from 20 to 100 J are shown in Figure 6.

Figure 6 elucidates that the contact force histories predominantly resemble the shape of a mountain (steep on the left while gentle on the right), and the peak force is approximately 4300 N, indicative that the loading time of the single GFRP sheet is obviously less than the unloading duration under different impact energies. Initial segments across all specimens exhibit a notable smoothness, succeeded by an oscillatory phase in the contact force curve and a subsequent smooth unloading segment post peak-force attainment, marked by a sharp decline in contact force and subsequent intense oscillations, indicative of damage to the specimens.

**Figure 7:**
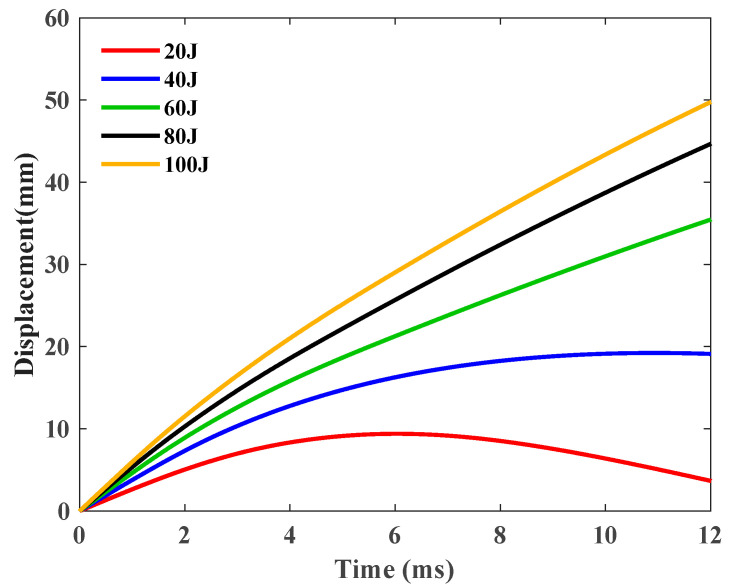
Displacement–time curves of the GFRP sample under 20 J, 40 J, 60 J, 80 J, and 100 J.

All generated tip displacement–time curves of the GFRP tests are summarized in Figure 7.

The displacement history was deduced from the recorded contact force data, the mass of the impactor, and the initial velocity of the impact for each test conducted. The chronology of the tip displacement was computed through a double integration of the force–time record, as per the equation delineated below:

(9) $$D=\int v_{0}dt+\iint \frac{F(t)-Mg}{M}d^{2}t$$

where D is the tip displacement; F(t)  is the force acquired by the data acquisition system; M is the total mass of the impactor (5.5 kg); $v_{0}$ is the touch velocity calculated by initial height of the drop weight; and g is the gravitational acceleration—9.8 $m/s^{2}$.

Under the influence of an impact energy of 20 J (Figure 7), the displacement initially escalates almost linearly, subsequently decelerating before it reaches its zenith. Following the apex, the displacement commences its decline, signifying the rebound of the impactor, attributable to the elastic resilience inherent in the GFRP material specimens. The remaining quartet of specimens exhibit analogous displacement history curves. When subjected to an impact energy of 40 J or beyond, a novel displacement–time curve manifests, wherein the displacement ascends almost linearly to the maximum devoid of any regression, insinuating that the impactor has perforated either the top or bottom plate of a specimen. In addition, akin to the contact force data, the displacement intensifies more precipitously with the surge in impact energy.

#### 4.1.2. Contact Force vs. Displacement Response

**Figure 8:**
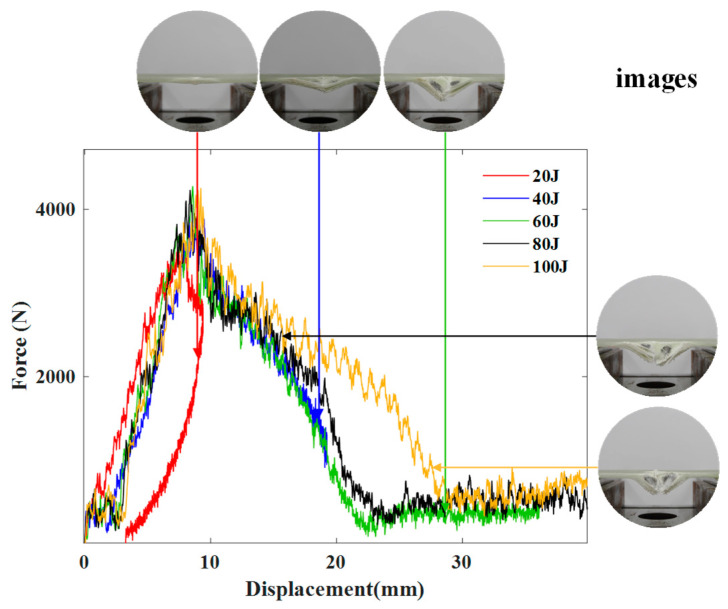
Contact force–displacement curves of the GFRP sample under different energies and images after impact.

In order to investigate the change of the strength of the specimens during the impact process, we made force–displacement diagrams. The contact force–displacement curves corresponding to each energy level for all tested simple GFRP specimens are compared in Figure 8.

Figure 8 shows the comparison between the force–displacement responses of the GFRP sample under 20 J, 40 J, 60 J, 80 J, and 100 J. As shown in Figure 8, the impact energy shows little influence on the initial stiffness, as its force–displacement curves show a highly consistent slope in the ascending section of loading. However, the damage due to impact is more visible as the impact energy grows. Additionally, the corresponding post-test damage states reveal an unequivocal trend of escalating damage—commencing from localized damage, such as dents, matrix cracks, and minor delaminations, and evolving to composite damage, characterized by extensive delaminations and fiber breakage. Under 20 J, the GFRP plate withstood the impact. When the impact energy exceeds 40 J, the force–displacement curves show a same trend of the GFRP specimens being penetrated.

#### 4.1.3. Evolution of Impact Energy and Its Absorption

**Figure 9:**
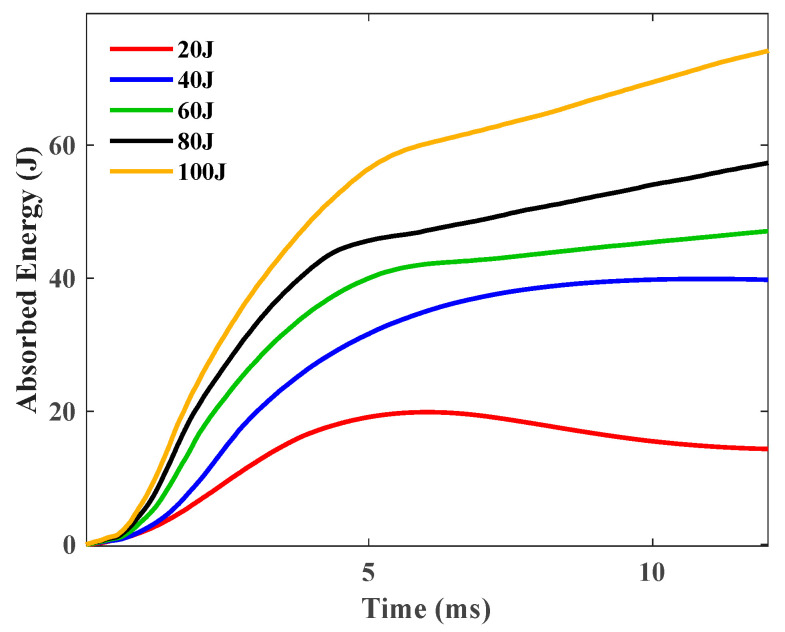
Energy absorption histories of the GFRP sample under 20 J, 40 J,60 J, 80 J, and 100 J.

The absorption of energy during an impact is a cardinal parameter for scrutinizing the dynamic response of a sandwich structure. The energy absorbed by a sandwich structure specimen is embodied by the area confined within a contact force versus displacement curve. Consequently, the value of energy absorption can be computed by integrating the contact force–displacement curve in the following manner:

(10) $$E_{absorbed\;}=\int F(\delta )d\delta$$

where $E_{absorbed}$ represents the absorbed energy; F(δ) the contact force–displacement relationship; and δ the displacement. The variation in the absorbed energy in real-time of simple GFRP plate specimens is depicted in Figure 9.

The energy transference from the impactor to the composite specimens, culminating in the absorption of energy at the conclusion of impact events, escalates in tandem with the impact energy. This is attributable to the fact that a heightened impact energy inflicts more severe damage upon a composite specimen. Only the 20 J impact has seen a significant pullback reflected the rebounding of the impactor.

### 4.2. Impact Responses for Sandwich Structures

#### 4.2.1. Contact Force and Tip Displacement Histories

**Figure 10:**
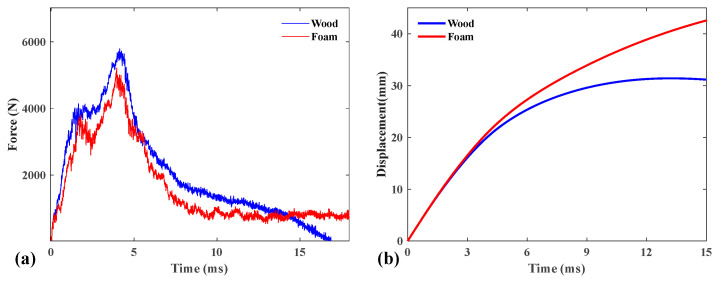
(**a**) Contact force and (**b**) tip displacement histories of the sandwich structure with different cores under 100 J.

As described above, the raw data obtained from the drop hammer impact device are expressed as a time axis of the contact force. The contact force and tip displacement histories of the sandwich structure with different cores under 100 J are shown in Figure 10.

The contact force histories during these low-velocity impact tests were documented and are represented in Figure 10a. The contact force–time trajectories demonstrate a linear increase, with their gradients exhibiting similar characteristics. Specifically, the gradient for the cork wood core sandwich laminate is a little bit more pronounced than that for the PS foam core sandwich laminate and experienced more oscillations during the impact. All generated tip displacement–time curves of the two sandwich panels are shown in Figure 10b. The calculation of displacement history is based on the same approach as that described in the previous section. As evidenced, they exhibit analogous curves, especially for the initial stage during the impact.

#### 4.2.2. Contact Force vs. Displacement Response

**Figure 11:**
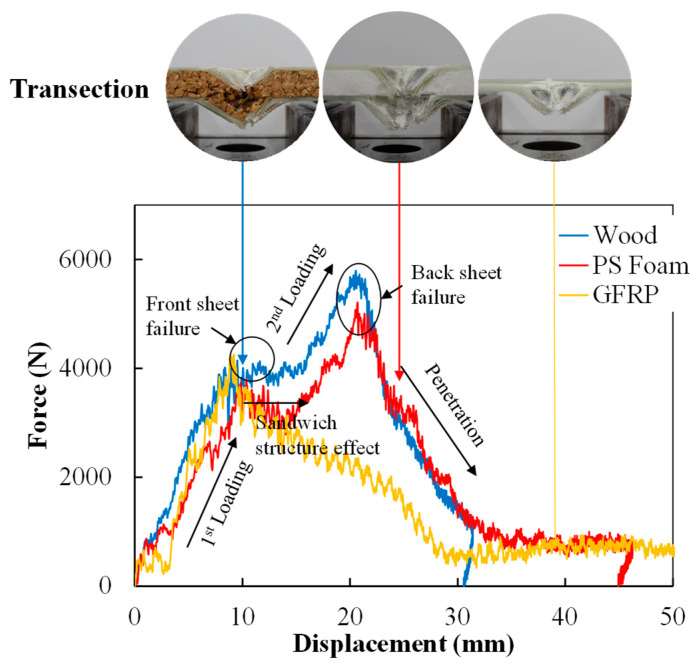
Contact force–displacement curves of the sandwich structure with different cores under 100 J and cross-sectional images after impact.

The contact force–displacement curves corresponding to the different cores of the sandwich structure for all tested specimens are compared in Figure 11. For comparison, the contact force vs. tip displacement curve for a single GFRP specimen is also displayed.

Distinct types of force–displacement curves signify different failure modes. The relationship between contact force and displacement is deemed a crucial hallmark of a material’s reaction to impact loading. It can be seen that, as the impact energy escalates to a certain threshold, failure of the front panel becomes apparent. This is marked by a precipitous drop in force after reaching the peak (all around 4250 N), succeeded by a considerably extended and intense oscillation phase. This phenomenon can be aptly termed the ‘sandwich structure effect’. It represents the damage deformation and a subsequent process of internal stress rebalancing. It also reveals that the sandwich structure (compared with the pure GFRP plate) possesses notable toughness. Under 100 J impact energy, a more pronounced peak is observed after an extended oscillation period (sandwich structure effect), indicating that the impactor has penetrated the fiber layer and made contact with the back plate. It can be seen that the peak forces that the sandwich panels can withstand has increased significantly compared to that of a single GFRP, with a 22.7% increase for the PS foam core sandwich panel and a 36.3% for the wood core sandwich panel. In the final descending section, a relatively smooth unloading phase can be discerned. It is clear that, due to the presence of sandwich structures, more impact energy is absorbed.

**Figure 12:**
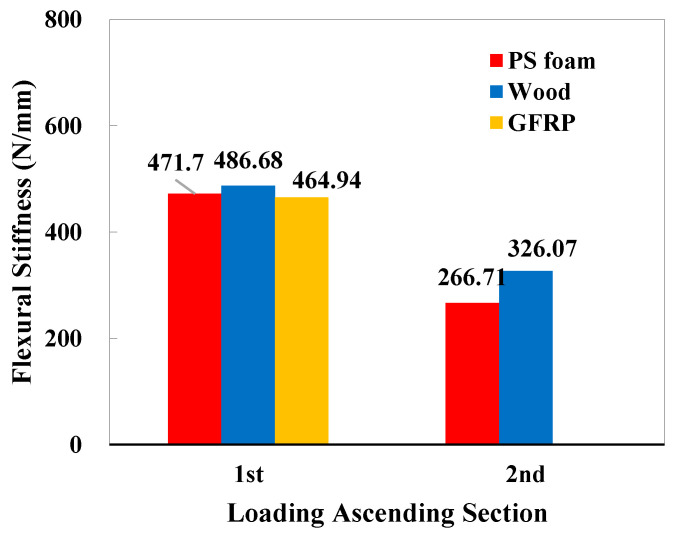
Comparison of the initial (1st) and secondary (2nd) flexural stiffness of sandwich panels via the original slope of the force–displacement curve in Figure 11.

According to Atas et al. [37], Wang et al. [38], and Rahman et al. [39], the slope of the ascending section of force–displacement curves represents the flexural stiffness of composite plates under impact loading. Specifically, due to the sandwich structure effect, both types of sandwich panels exhibit a second ascending segment. A comparison to the stiffness was presented using the slope of the moving average of the force–displacement curves (with 100 J impact), which is depicted in Figure 12. It can be seen that the initial stiffnesses (1st) are quite similar, with a difference of no more than 5%. However, due to the differences in core materials, there is a significant disparity in the equivalent flexural stiffness of the second ascending segment, with nearly a 22.3% difference.

**Figure 13:**
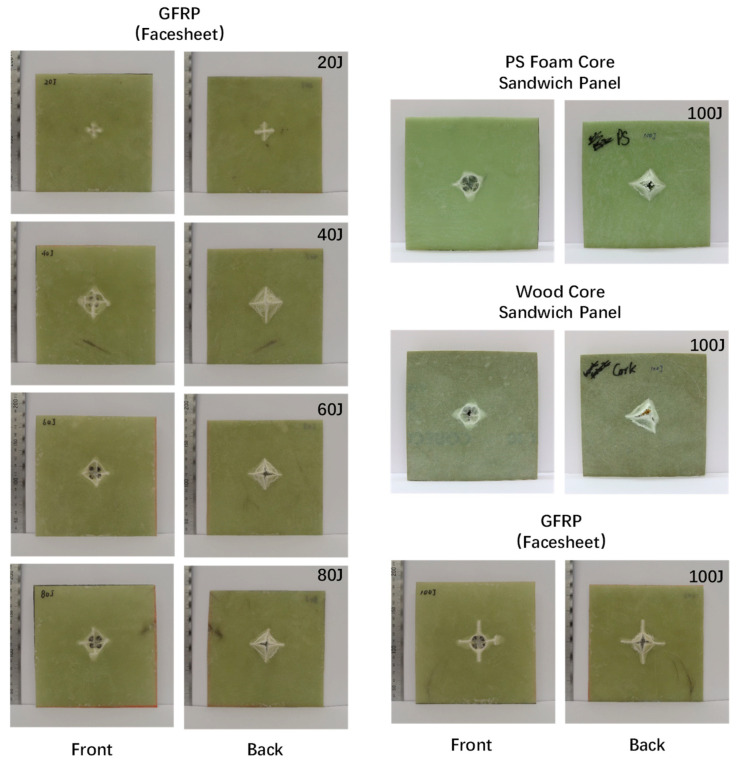
Comparison of the post-test damage status on both front and back surfaces of all tested specimens.

Figure 13 illustrates a comparison of the post-test damage status on both the front and back surfaces of all tested specimens. The crack lengths on the front and back faces of the specimens are summarized in Table 2. Overall, the figures of force–displacement, displacement histories, force histories, and energy absorption histories have the same trends and characteristics as those in the study by Sun et al. [17], further confirming the credibility of the results.

### 4.3. Validation of the Finite Model

**Figure 14:**
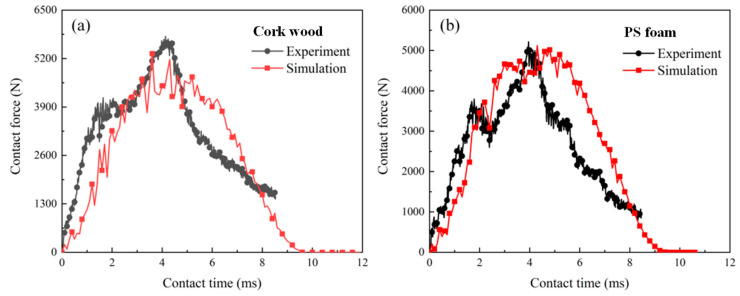
Contact force history from experiment and simulation results: (**a**) sandwich structure with cork wood core; (**b**) sandwich structure with PS foam core.

The contact force from the experimental results and numerical results are compared in Figure 14. The detailed values are listed in Table 3. For the sandwich structure with a wood core, the contact peak force from the experimental results and numerical results are 5794 N and 5329 N; the relative error is 8%. For the sandwich structure with a PS foam core, the contact peak force from the experimental results and numerical results are 5794 N and 5329 N; the relative error is 18%. The numerical model can reflect the impact mechanical response to a certain extent. The sandwich structure with cork wood core exhibited a higher contact force than that with a PS foam core.

### 4.4. Comparison of Damage Initiation and Evolution

**Figure 15:**
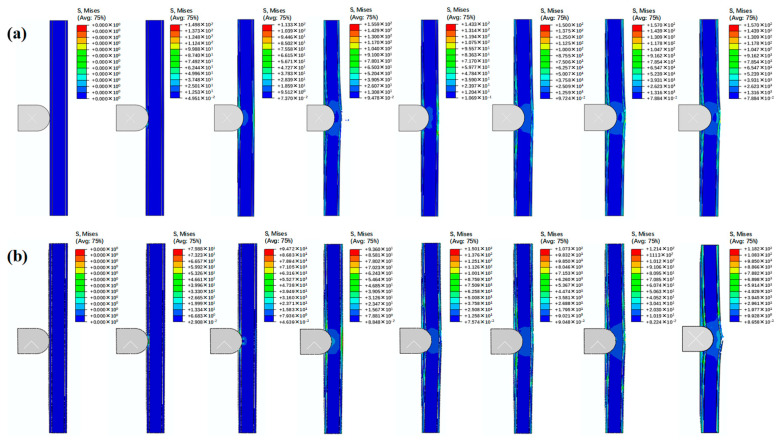
Failure process of sandwich structures with (**a**) cork wood core and (**b**) PS foam core.

The failure processes of sandwich structures with different cores are shown in Figure 15. For the sandwich structure with a wood core, the stress concentration initially occurred at the impact point on the impact surface. Then, the stress is transmitted to the impact point on the back surface. The composite panel showed some damage, and the core exhibited obvious plastic deformation and cracks. With the impact progress, the composite panel on the back surface of the sandwich structure appears to have failed. For the sandwich structure with a wood core, a similar failure process was observed. The damage initially occurred at the impact point on the impact surface. Then, the core showed plastic deformation and damage. Finally, the composite panel on the back surface failed.

**Figure 16:**
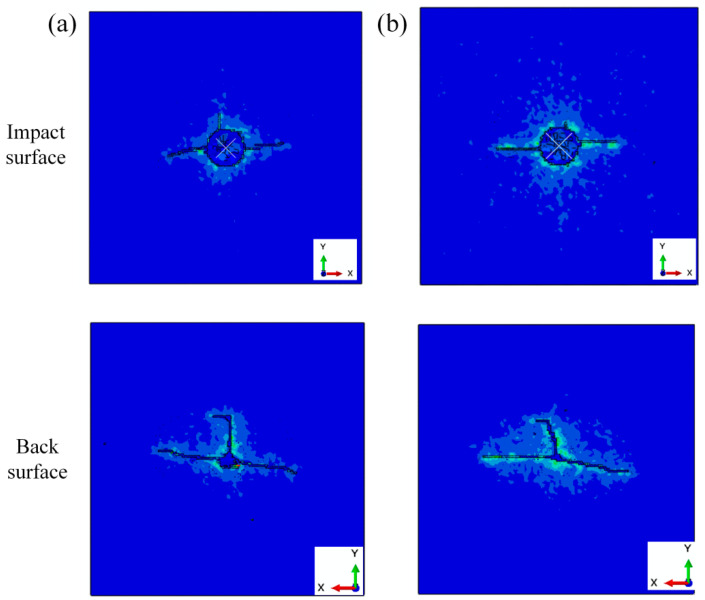
Failure pattern of sandwich structures with (**a**) cork wood core and (**b**) PS foam core.

The failure patterns of sandwich structures with different cores are shown in Figure 16. For the impact surface, the impact surfaces were fragile at the impact point. The cracks through the impact point are observed on the impact surface. For the back surface, cracks in a T-shape were observed on the back surface of the sandwich structure. There were also some fragile areas at the impact point. The fragile areas at the back surface were much smaller than those on the impact surface.

## 5. Summary and Conclusions

The impact behaviors of sandwich structures with PS foam cores and cork wood cores were analyzed and compared through experimental and numerical methods.

The experimental results and data analysis proved that the sandwich structure is superior to the single GFRP structure in terms of strength, stiffness, and energy absorption. This shows that the sandwich structure is an effective measure to improve the properties of composite materials.

The contact force, failure pattern, and failure process were compared for sandwich structures with different cores. The results showed that the contact force of the sandwich structure with the wood core was higher than the foam core. For both sandwich structures, the damage initially occurred at the impact point on the impact surface. The core showed plastic deformation and damage. The failure pattern showed that there were fragile areas at the impact point on the impact surface.

The accurate numerical model will help designers to perform a parametric study that will help to choose an appropriate core material that will maximize the impact-resistance capability of a sandwich structure.

Based on the current results, sustainable core materials, such as cork wood, can serve as a reliable alternative to the commonly used polystyrene (PS) foam core in sandwich structures. Apart from these significances, there are only two core layers in this group of experiments, and there is not enough coverage. Therefore, the conclusions drawn have certain limitations, which are expected to be improved in subsequent research.

## Figures and Tables

**Table 1:** Material properties of sandwich panel constituent materials.

Materials		Face-Sheet (E-Glass) [22,34,35]	Wood (Cork Core) [22,32,33]	Foam (Polystyrene (PS) Core) [22,33,36]
Density (kg/m^3^)	*ρ*	1850	150.4	32.4
Young’s Modulus (GPa)	*E1*	27.1	0.013	0.016
	*E2*	27.1	0.013	0.016
	*E3*	12	0.021	0.016
Poisson’s Ratio	*υ* *12*	0.11	0.1	0.3
	*υ* *23*	0.18	0.1	0.3
	*υ* *31*	0.18	0.1	0.3
Shear Modulus (GPa)	*G12*	2.9	0.0044	0.0025
	*G23*	2.14	0.0044	0.0025
	*G31*	2.14	0.0044	0.0025
Strength (MPa)	tensile	604 (in-plane), 58 (out-of-plane)	0.6	0.5
	compressive	291	0.3	0.3
	shear	75 (matrix), 85 (fiber)	5.9	4.5

**Table 2:** Crack lengths on the front and back faces of the tested specimens.

Specimen	Front_x ^1^ (mm)	Front_y ^2^ (mm)	Back_x (mm)	Back_y (mm)
GFRP_20 J	17.98	15.65	25.7	26.25
GFRP_40 J	28.76	31.53	45.92	42.95
GFRP_60 J	33.74	32.32	41.75	43.01
GFRP_80 J	35.67	35.88	44.53	43.99
GFRP_100 J	51.88	50.76	57.94	57.82
PS foam_100 J	41.08	39.83	49.76	41.08
Wood_100 J	42.32	33.61	43.56	44.81

^1^ Horizontal directions of the image plane, and ^2^ vertical directions of the image plane, in Figure 13.

**Table 3:** Contact peak force value from experiment and simulation results.

	Cork Wood Core	PS Foam Core
Experiment contact force (N)	5794	5213
Simulation contact force (N)	5329	5120

## Data Availability

The original contributions presented in this study are included in the article. Further inquiries can be directed to the corresponding authors.
